# The Role of Apoptosis in the Pathology of Pancreatic Cancer

**DOI:** 10.3390/cancers3010001

**Published:** 2010-12-23

**Authors:** Nicole Samm, Kristin Werner, Felix Rückert, Hans Detlev Saeger, Robert Grützmann, Christian Pilarsky

**Affiliations:** Department of Visceral-, Thoracic-and Vascular-Surgery, University Hospital Dresden, Dresden, Germany; E-Mails: sammynic82@yahoo.de (N.S.); Kristin.Werner@uniklinikum-dresden.de (K.W.); Felix.Rueckert@uniklinikum-dresden.de (F.R.); Hans-Detlev.Saeger@uniklinikum-dresden.de (H.D.S.); Robert.Gruetzmann@uniklinikum-dresden.de (R.G.)

**Keywords:** apoptosis, signal transduction pathway, pancreatic cancer

## Abstract

Pancreatic cancer is a disease with high resistance to most common therapies and therefore has a poor prognosis, which is partly due to a lack of reaction to apoptotic stimuli. Signal transduction of such stimuli includes a death receptor-mediated extrinsic pathway as well as an intrinsic pathway linked to the mitochondria. Defects in apoptotic pathways and the deregulation of apoptotic proteins, such as Survivin, Bcl-2, Bcl-x_L_ and Mcl-1, play decisive roles in the development of pancreatic cancer. Investigation of the molecular mechanism allowing tumors to resist apoptotic cell death would lead to an improved understanding of the physiology and the development of new molecular strategies in pancreatic cancer.

## 1. Introduction

Pancreatic cancer is one of the most malignant and aggressive types of cancer in humans and carries a very poor prognosis. With 40,000 new cases diagnosed in the United States each year, pancreatic cancer is the fourth and fifth leading cause of cancer-related death in the Western world for males and females, respectively [1]. Approximately 95% of exocrine pancreatic cancer cases are ductal adenocarcinoma (PDAC) [2]. In 65% of these cases, the tumor is located in the head of the pancreas; in 30%, in the corpus; and in 5%, in the tail of the organ [3]. The delayed appearance of symptoms causes a late diagnosis; as a result, roughly 85% of patients show an organ overlapping growth of the tumor when the disease is first discovered, and only the remaining 15% of patients have an opportunity for curative surgical treatment. Despite improvements made in surgical techniques and pre- and postoperative care, less progress has been seen in improving the survival of patients with this type of cancer [3]. The five-year survival rate after surgical resection is about 20% and is about 5% for all patients [1]. Furthermore, uncontrolled proliferation, a high metastatic potential and resistance to most adjuvant therapies also contribute to the very poor prognosis of this disease. The response to oncology therapy options such as chemotherapy, radiotherapy and immunotherapy is not satisfying. Tumor development and progress of PDACs as well as resistance to most oncology therapies involve the absence of a reaction to apoptotic stimuli [4].

Resistance to apoptosis and the ability to evade this process are two of the hallmarks of human cancer [5]. This review will discuss the facts about apoptotic pathways and the deregulation of apoptotic proteins in pancreatic cancer to demonstrate the correlation between disease occurrence and defects in apoptotic mechanisms.

## 2. Apoptosis—A General Overview

In 1842, Carl Vogt described the concept of natural cell death for the first time [6]. Since 1972, Kerr, Wyllie and Curry have been linked to the word apoptosis and the development, progression and treatment of cancer. They published a paper in which they characterized and defined this form of cell death and gave birth to the term apoptosis [7,8]. The word apoptosis is of Greek origin and refers to the fall of leaves in autumn.

Apoptosis is an intrinsic cell suicide program. As a central regulation mechanism for tissue homeostasis, it is involved in the regulation of many physiological and pathophysiological processes such as the differentiation of the embryonic body figure, development of the nervous system, formation of the immune system and the homeostasis of the number of cells in proliferating tissue [9,10].

In contrast to necrosis, apoptosis takes place without any inflammatory reaction and is marked by cellular shrinking, condensation of the chromatin and ruffling of the plasma membrane with loss of contact to other cells of the cell assembly [11,12]. It is described as a breaking-up of the cell into apoptotic bodies. These bodies, which consist of cell organelles and nuclear material, are surrounded by an intact plasma membrane. Macrophages recognize the apoptotic cell fragments and abolish them by phagocytosis [13].

**Figure 1.**
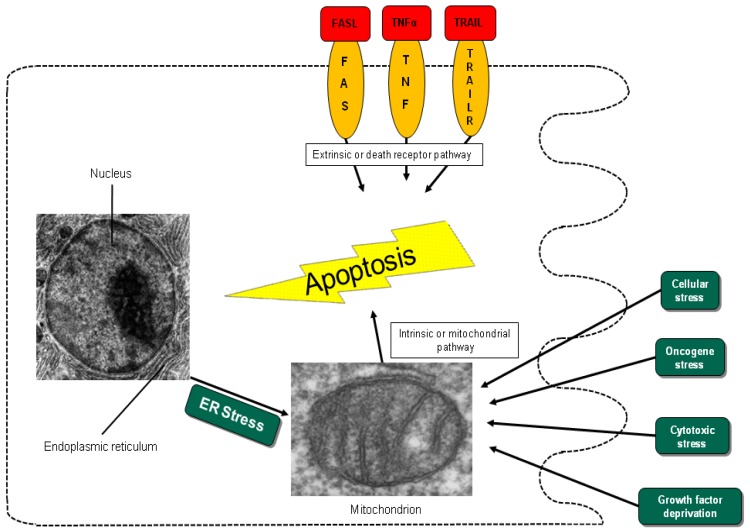
Different entry points to apoptosis. The extrinsic or death receptor pathway is triggered by the interaction of the receptors Fas, TNF and TRAIL and their natural ligands. The intrinsic or mitochondrial pathway is activated by factors such as DNA damage, cytotoxic stimuli or cell stress. Another special entry point to intrinsic apoptosis begins in the endoplasmic reticulum, where internal variability within the cell, e.g., variation in calcium homeostasis or wrongly folded proteins, leads to the initiation of apoptosis [14,15].

Different entry points to apoptosis have been described, such as the death receptor-mediated or extrinsic pathway and the mitochondrial intrinsic pathway [12]. Another entry point for intrinsic apoptosis has been identified at the endoplasmic reticulum (ER) (Figure 1) [15,16]. Cell irritation, such as variation in calcium homeostasis or a collection of wrongly folded proteins, causes stress that initiates apoptosis by activation of the unfolded protein response or cleavage of the cargo receptor protein BAP31 [15,17,18]. This mechanism causes the transfer of calcium from the endoplasmic reticulum into the mitochondria and the initiation of cytochrome c release [15]. All pathways converge in the activation of the executive cell death enzymes, the effector caspases 3, 6 and 7. These enzymes conclude terminal apoptosis by cleaving the nuclear lamina, DNase inhibitors and cytoskeletal proteins.

These complex pathways are controlled and affected by an array of different pro- and anti-apoptotic factors, which are important in ensuring tissue balance [12]. Altered expression levels and mutations that influence the activation and function of these pro- and anti-apoptotic genes influence cancer cell sensitivity to chemotherapy, radiotherapy, tumor development and progression [9].

## 3. The Extrinsic Pathway

**Figure 2.**
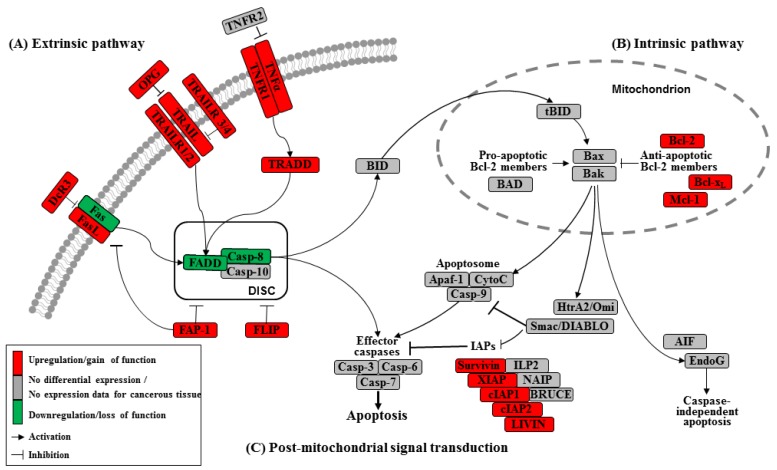
A map of the molecular mechanisms of apoptosis [19]. Apoptosis pathways can be initiated by different stimuli. The left side shows death receptor ligation at the plasma membrane (**A**), and the right side shows activation at the mitochondria (**B**). Stimulation of the death receptor results in receptor aggregation and recruitment of the adaptor molecule FADD and caspase 8. Upon recruitment, caspase 8 becomes activated and initiates apoptosis by the direct cleavage of downstream effector caspases. The intrinsic pathway can be initiated in mitochondria by stress stimuli and is regulated by the balance of the action of pro-apoptotic and anti-apoptotic Bcl-2 protein family members. (**C**) Inhibitor of apoptosis proteins (IAPs) and other signal transduction molecules are able to diminish the effect of caspase 8 activation.

Apoptosis can be initiated by extrinsic but endogenous “death signals”. The receptors TNF (tumor necrosis factor), Fas (Apo-1, CD95) and TRAILR (TNF-related apoptosis-inducing ligand, Apo-2) are members of the TNF receptor superfamily and share a common internal death domain. Activation occurs by their natural ligands TNFα, FasL (Fas-ligand) and TRAIL [20]. The interaction between receptor and ligand causes trimerization of the receptor followed by the activation and recruitment of FADD (Fas-associated death domain protein) and procaspases 8 and 10. This activation subsequently initiates the formation of the death-inducing signaling complex (DISC) (Figure 2) [21]. The death domain that forms the DISC attaches to procaspase 8, which has low proteolytic activity. Through this connection to DISC, the local concentration of procaspase 8 increases, leading to an autoproteolytic cleavage and the release of active caspase 8. By cleavage next to their aspartate residues, the caspases activate each other, leading to a caspase cascade. Finally, the effector caspases 3, 6 and 7 conclude terminal apoptosis by cleaving the nuclear lamina, DNase inhibitors or cytoskeletal proteins [9,22,23].

## 4. The Death Receptor

The death receptor Fas is part of one of the main apoptotic cell death signaling pathways (Figure 2) [23]. Altered expression levels of Fas or FasL have been found in many human cancers. Reports concerning Fas receptor expression in pancreatic cancer are conflicting. It has been demonstrated that Fas mRNA levels are elevated in pancreatic carcinomas [23]. However, native pancreatic tumor cells show very little Fas receptor expression, though they express high levels of FasL and its antagonistic decoy receptor DcR3. Decoy receptors can also bind these death ligands, but because of their missing death domain, they do not transduce the apoptotic signal, thereby preventing this ligand from associating with the conventional signal receptor. Altogether, these data indicate that tumor cells, especially those from pancreatic carcinoma, can evade Fas-mediated apoptosis by downregulation of the Fas receptor [23,24]. This resistance is also closely linked to the expression of FAP-1 (Fas associated phosphatase-1) and several other intracellular proteins such as Bcl-2, Bcl-x_L_ and FLIP [25,26].

The protein tyrosine phosphatase FAP-1 appears to be overexpressed in pancreatic cancer cells and protects these cells from Fas-mediated apoptosis by inhibiting the activation of caspase 8 (Figure 2) [26,27]. FAP-1 can interfere with the translocation of Fas to the cell surface. This interference causes a low receptor density and an interruption of receptor trimerization, which would be essential for DISC formation. In addition, FAP-1 may inhibit caspase 8 in a direct manner [20,26].

Another strong inhibitor of caspase 8 activation is FLIP (Figure 2). FADD-like ICE inhibitor proteins (FLIPs) are structural homologues of caspase 8 and compete with procaspase 8 for binding to FADD at the DISC. The proteins of this family are highly expressed in pancreatic carcinoma cells, resulting in a suppressed signal transduction [28-30]. In addition to high expression of FLIP, a low quantity of FADD was shown to be a factor important for resistance against FasL- and TRAIL-induced apoptosis in pancreatic carcinoma [20,24], which might cause the progression of malignant pancreatic carcinoma.

A second death receptor system recognizes the tumor necrosis factor α (TNFα) (Figure 2), an inflammatory cytokine with a large variety of biological functions such as regulation of cell death and survival, differentiation and inflammation. There are two different kinds of TNF receptors: TNF receptor 1 (TNFR1) is responsible for signal transduction, while TNF receptor 2 (TNFR2) is a decoy receptor [31]. TNFR1 and TNFR2 are not overexpressed in pancreatic cancer; therefore, no participation in the mechanism of apoptosis resistance is anticipated [32,33].

A third extrinsic stimulus is provided by TRAIL (Figure 2). Identified in 1995, TRAIL has two death receptors, TRAIL-R1/DR4 and TRAIL-R2/DR5, and three antagonistic decoy receptors, TRAIL-R3/DcR1, TRAIL-R4/DcR2 and Osteoprotegrin (OPG) [34]. TRAIL is normally expressed by natural killer cells in the immune system to combat tumorigenesis. In pancreatic cancer cells, TRAIL receptors as well as the decoy receptors TRAIL-R4 and OPG are highly expressed [35], and it appears that the TRAIL system is functional in pancreatic cancer but is blocked at apoptotic pathways downstream [36].

**Figure 3.**
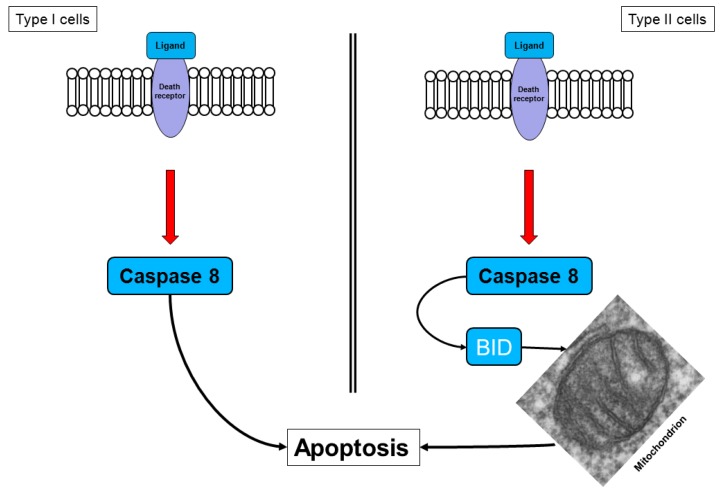
Apoptosis mediated by death receptors in type I and type II cells. In type I cells, the quantity of initiator caspases is adequate to induce apoptosis directly, whereas in type II cells, the enhancing effect of mitochondria is necessary.

Two different cell types have been identified for the death receptor signaling pathway (Figure 3). In type I cells, the death receptor complex, together with the adaptor molecule FADD, recruits procaspase 8, which is then cleaved to the active caspase 8, thereby activating the effector caspases [37]. This action implies that, in type I cells, the activated initiator caspases are sufficient to induce executioner caspases directly.

Pancreatic cancer cells are type II cells [27,37]. These cells have reduced receptor complex formation; thus, less procaspase 8 can be activated (Figure 3). Signal amplification is required for apoptosis induction. In these cells, caspase 8 cleaves BID (a Bcl-2 family member), which translocates to the mitochondrial membrane and induces the release of apoptotic factors [27,37]. The signal requires the enhancing effect of the mitochondria to induce apoptosis, a mechanism that is called the mitochondrial amplification loop [20,22].

## 5. The Intrinsic Pathway

In the intrinsic or mitochondrial pathway of apoptosis, caspase activation is closely connected to the permeabilization of the outer mitochondrial membrane. This is mediated by pro-apoptotic members of the Bcl-2 family, mitochondrial lipids, proteins that control bioenergetic flux and components of the permeability transition pore [38]. Numerous cytotoxic stimuli and pro-apoptotic signal-transducing molecules affect the permeability of the outer mitochondrial membrane (Figure 1). Trigger points or intracellular signals for the activation of the mitochondrial pathway include DNA damage, oxidative and cytotoxic stresses and ER stress [39]. By disrupting the outer membrane, a collection of proteins normally found in the space between the inner and outer mitochondrial membranes are released; these proteins include cytochrome c, apoptosis inducing factor (AIF), SMAC/DIABLO, Omi/HtrA2 and endonuclease G [40]. In the cytosol, these apoptotic proteins initiate cell death by promoting caspase activation [11]. The release of cytochrome c triggers caspase 3 activation by formation of the apoptosome complex, which contains cytochrome c/Apaf-1/caspase 9 (Figure 2) [41,42]. The construction of the apoptosome and the release of cytochrome c seem to be unperturbed in pancreatic cancer cell lines [43]. SMAC/DIABLO and Omi/HtrA2, which show normal expression levels in pancreatic carcinoma cells [44], enable caspase activation by neutralizing important endogenous inhibitor of apoptosis proteins (IAPs) [45-47].

Most of the cell death in human cells is initiated by the intrinsic pathway and results from an unregulated increase in mitochondrial membrane permeability [48]. Pathologic alterations of the intrinsic pathway in pancreatic cancer include intramitochondrial signal transduction, which is regulated by the Bcl-2 protein family [49,50]. It is the comparative balance of these pro- or anti-apoptotic members of this family that determines if activation of the intrinsic pathway occurs.

Links between the receptor (extrinsic) and the mitochondrial (intrinsic) pathway exist at different levels of the machinery. BID (BH3-interacting domain death agonist) is a pro-apoptotic member of the Bcl-2 protein family with a BH3 domain. Once cleaved by caspase 8, it translocates as “truncated BID” (tBID) to the mitochondrion where, along with other pro-apoptotic Bcl-2 proteins, it initiates the intrinsic pathway (Figure 2) [51,52]. Type II cells, which have low caspase 8 activation as seen in pancreatic carcinoma cells [37], can achieve this mitochondrial amplification loop for an efficient transduction of the apoptotic signal [21].

In summary, the death program can be initiated by different intracellular or extracellular stimuli that activate the common cell death machinery downstream. Both pathways result in activation of the effector caspases, which cleave proteins that are very important for the rigidity and function of the cells.

## 6. Regulation of Apoptosis

The Bcl-2 gene was discovered in 1986 by Yoshide Tsujimoto at the junction of the hallmark t(14;18) chromosome translocation characteristic of human follicular lymphoma [53]. It was named Bcl-2 for B-cell lymphoma 2 [54].The expanding number of proteins related to Bcl-2 by sequence homology and participation in the control of the apoptotic machinery has led to the definition of a Bcl-2 family of proteins [55].

The Bcl-2 family has two pro-apoptotic subgroups, the BAX-like subgroup (BAX, BAK and BOK) and the BH3-only subgroup (BAD, BIK, BID, BIM, BMF, HRK, NOXA and PUMA). The only anti-apoptotic group consists of Bcl-2, Bcl-x_L_ and Mcl-1 (Figure 2) [22,38]. Bcl-2 family proteins interact with other molecules through an α-helical domain termed the BH-3 domain [48]. The BH3-only proteins act as sensors of cellular stress, directly antagonizing anti-apoptotic Bcl-2 members and activating BAX-like proteins, ultimately leading to permeabilization of the outer mitochondrial membrane [56,57].

Bcl-2, an anti-apoptotic family member is located on the cytoplasmic side of the mitochondrial outer membrane, at the ER-membrane or on the nuclear cover. It registers damage to these compartments and prevents the release of cytochrome c from mitochondria in a number of different tissues [58]. In a few human tumors, high expression of Bcl-2 has been found, but the expression in pancreatic cancer cells is normal or even decreased [57,59].

Bcl-x in human cells is present in two distinct isoforms. Bcl-x_L_ is the longer form, acting in an anti-apoptotic manner. Bcl-x_S_, the shorter form, performs as an apoptosis promoter, in contrast to Bcl-x_L_. It has been shown that Bcl-x_L_ prevents the release of cytochrome c from the mitochondria [48]. In type II cells such as pancreatic cancer cells, the overexpressed Bcl-x_L_ plays the most important role in protecting the cell from Fas-and TRAIL-mediated apoptosis [35].

Another anti-apoptotic Bcl-2 family member that is highly overexpressed in pancreatic cancer cells is Mcl-1. High concentrations of this protein protect the cancer cells from hypoxia and oxidative stress during tumorigenesis [20]. Fritsch *et al.* showed rapid and selective downregulation of Mcl-1 that preceded the activation of BAX, BAK and caspases [60].

Of the pro-apoptotic members of the Bcl-2 family, Bax (Bcl-2 associated X protein) resides in the cytosol and translocates to mitochondria upon induction of apoptosis [61]. Overexpression of Bax does not influence the apoptosis rate or expression of Bcl-2 and Bcl-x_L_ in human pancreatic cancer cells transduced with a retroviral expression vector [62]. In pancreatic cancer, Bak (Bcl-2 antagonist/killer protein) expression and apoptosis occur in regions of chronic inflammation surrounding the cancer cells but not in the tumor cells themselves, which may simplify accelerated growth and spread [63]. Bad, a typical pro-apoptotic member of the Bcl-2 family, binds with its BH3 domain to both Bcl-2 and Bcl-x_L_ and mediates the pro-apoptotic function of Bcl-x_L_ [64].

In summary, intramitochondrial signal transduction in pancreatic cancer cells is unbalanced towards the anti-apoptotic side [50,65,66]. The deregulation of both the pro- and anti-apoptotic Bcl-2 proteins plays a crucial role both in the development, growth and expansion of pancreatic cancer and in the resistance to current therapy options [37,55].

## 7. Main Mediators of Apoptosis

Caspases (Cysteine-dependent aspartate specific proteases) are proteases that contain a cysteine in their active center and are able to cleave proteins, particularly behind an aspartate residue [46]. Based on their pro-apoptotic functions, the caspases have been divided into two groups. The initiator caspases 2, 8, 9 and 10 are at the beginning of the signal cascade and are involved in the initiation of apoptosis. The effector or downstream caspases 3, 6 and 7 cleave their substrates; this action leads to cell death because the caspases are able to degrade multiple substrates directly, including structural and regulatory proteins in the cell nucleus, the cytoplasm and the cytoskeleton (Figure 2) [67].

In healthy cells, all of the caspases are constantly expressed in their inactive form as proenzymes in the cytoplasmic milieu. They are synthesized as a single chain of inactive zymogens composed of four domains: an N-terminal prodomain of variable length, a large subunit with a molecular weight of about 20 kDa, a small subunit and a linker region connecting these catalytic subunits [67,68]. Activation during progression of apoptosis happens by a proteolytic processing. Caspases initiate and execute cell death by inactivating anti-apoptotic proteins, shutting down DNA replication and repair and disrupting the cytoskeleton and nuclear lamina [37].

Caspase 8 is essential for the extrinsic cell death pathway, which is initiated by TNF family members. Death receptors recruit the DISC upon binding specific TNF family ligands and trimerization. Several reports show that caspase 8 is mutated in diverse types of cancer, especially gastric cancer [69-71].

Caspase 9, the apical initiator caspase within the apoptosome-dependent cascade, is an almost ubiquitous protease. The activated apoptosome binds caspase 9 and activates the enzyme and caspase 3 downstream. It is expressed constitutively in a variety of fetal and adult normal or cancerous tissues [41,72,73].

Caspase 3 is an effector caspase and can be activated by either the extrinsic or the intrinsic cell death pathway. It plays a central role in the execution phase of cell apoptosis. Several reports have focused on mutations of caspase 3 in a large group of different cancer types [74,75].

When the literature is analyzed for a correlation between these three main caspases in cancer development in general and pancreatic cancer in particular, it is clear that caspase expression is relatively normal in pancreatic cancer tissue. There is no evidence that a specific mutation of caspase 3, 8 or 9 leads to a higher incidence of pancreatic carcinoma [37,68].

Many different inhibitors and activators regulate caspase activation in a very strict manner. The oligomerization model explains that caspases exist as inactive monomers. The effector caspases bring them together, allowing for their intermolecular autoproteolytic activation. For example, to become functional, procaspase 8 requires association with its cofactor FADD, and procaspase 9 must interact with APAF-1 (Figure 2) [37,76].

In contrast to the normal expression of caspases in pancreatic cancer, effectors blocking caspase activation or function, such as FLIPs, show elevated expression in pancreatic cancer, leading to resistance to death receptor-mediated apoptosis [21,37].

## 8. IAP Family Inhibitors of Apoptosis Proteins

The family of inhibitor of apoptosis proteins (IAPs) was discovered in 1993 in the baculovirus genome [10]. There are eight human IAPs, including XIAP (X-linked inhibitor of apoptosis) and Survivin, which are direct inhibitors of caspases 3 and 7 and procaspase 9. Other members of this collective are cIAP 1 and 2 (cellular IAP1 and 2), ILP 2, ML-IAP, NAIP and BRUCE [45,77], which are involved in the signal transduction of human receptor complexes, for example TNFR2 (TNF receptor 2). Their expression is stimulated by growth factors and they are inhibited by mitochondrial proteins such as SMAC/DIABLO (second mitochondria-derived activator of caspase/direct IAP binding protein with low pI) (Figure 2). These proteins block caspases 3, 7 and 9 and lead to the ubiquitylation of caspases 3 and 7, which results in their degradation by the proteasome. IAPs are characterized by a 70–80 amino acid BIR domain (baculoviral IAP repeat), a carboxy-terminal zinc finger domain and one or more additional functional domains that are necessary for caspase interaction [78].

Overexpression of XIAP, cIAP1, cIAP2 and Survivin has been demonstrated to suppress apoptosis, but the cellular function of the IAP family members is unclear [78]. However, given their role in cellular homeostasis, it is not surprising that deregulation of IAP expression or function seems to be involved in a large number of cancer species [77]. Indeed, there are data suggesting that altered expression of cIAP1, cIAP2, XIAP and Survivin play a role in the pathogenesis of pancreatic cancer [37].

XIAP exerts an anti-apoptotic function by binding and inhibiting effector caspases such as caspases 3 and 7 and procaspase 9 [79,80]. It has been demonstrated that of all mammalian IAPs, XIAP is the only one that is truly a physiological inhibitor of caspases *in vivo* [81]. XIAP targeting has been shown to be required not only for effective induction of apoptosis but also for potent suppression of long term survival; this finding has important implications for the development of experimental strategies directed toward IAP proteins in human cancer, especially pancreatic cancer [45,57,79].

Survivin does not directly bind caspases but inhibits apoptosis by cooperative interactions with other partners *in vivo*. For example, such a partner for an IAP-IAP complex is XIAP [82]. Survivin expression is turned off during fetal development and is absent from non-neoplastic adult human tissues. With 142 amino acids, Survivin is the smallest mammalian IAP, and its structure contains only a single BIR domain and lacks a carboxy-terminal RING finger domain [37,83]. Survivin is expressed in the G2/M phase of the cell cycle. Survivin is a conspicuous cancer gene that is overexpressed in almost every human tumor, including neuroblastoma and cancers of the lung, colon, breast and prostate, whereas it is largely undetectable or expressed at very low levels in normal human tissues [82]. It has also been demonstrated that Survivin is frequently expressed in malignant pancreatic tumors [37]. Survivin is a potent caspase inhibitor whose overexpression in cancer cells is implicated in the resistance to different apoptotic stimuli, including resistance to therapy—especially chemotherapy—as well as disseminated disease and an overall inappropriate disease outcome [37,84]. Survivin appears to be situated at the junction of cell death and cell division, leading to a checkpoint involved in cytokinesis while also suppressing apoptosis [57,85].

IAPs are regulated by a protein complex named SMAC/DIABLO [37]. SMAC/DIABLO is synthesized as a precursor protein and is imported into the mitochondria by an N-terminal signal sequence. The aged form of SMAC/DIABLO is created via the cleavage of this signal [86]. In case of cellular stress, SMAC/DIABLO is released into the cytosol from the intermembrane space. The pro-apoptotic effect is mediated by its interaction with the inhibitors of caspases [87]. Release of SMAC/DIABLO from the mitochondria can be avoided by Bcl-2 [88]. Several studies have illustrated that overexpression of SMAC/DIABLO sensitizes neoplastic cells to apoptotic death. These findings have resulted in the development of small molecules fused to an N-terminal signal sequence; these molecules imitate the function of SMAC/DIABLO as therapeutic agents to induce death or to increase the apoptotic effects of chemotherapeutic agents [89-91]. For pancreatic carcinoma in particular, however, there is no evidence that SMAC/DIABLO plays a decisive role in avoiding apoptosis.

## 9. Conclusions

Apoptosis avoidance is one of the hallmarks of pancreatic cancer that promotes formation, progression and resistance to treatment. In this review, we have demonstrated that there are numerous molecular defects at multiple levels of the apoptotic pathway that lead to apoptosis resistance; these defects include the deregulation of death receptors, negative regulation of post-receptor signaling, the anti-apoptotic imbalance of the intramitochondrial Bcl-2 proteins and the upregulation of IAPs such as XIAP and Survivin. All of these expression changes, as well as mutations in apoptotic proteins, are commonly found in pancreatic cancer cells and lead to tumor development, tumor growth and metastasis. This deregulation of the apoptotic machinery may explain why pancreatic cancers are resistant to most adjuvant therapies, including immuno-, chemo- and radiotherapy.

A few innovative cancer therapy approaches, such as small molecule inhibitors (SMI) of Bcl-2 family proteins, have been described [92]. Another promising field of research is the design of TRAIL-based protocols that exploit the cytotoxicity of specific monoclonal antibodies to TRAILR1/R2 [93].

Nevertheless, these therapies represent only a fraction of the potential for future treatments. Therapies in the future should take advantage of all that is known about apoptosis and its role in cancer genesis. There is a need to combine numerous strategies to adapt the sensitivity of pancreatic cancer cells to apoptosis without affecting normal cells to enhance the prognosis or even cure patients of this aggressive form of cancer.

## Figures and Tables

**Table 1.** Compendium of the death receptors with their adequate ligands and decoy receptors. The terms in parentheses are the genes' aliases.

**Ligand**	**Death receptor**	**Decoy receptor**
FASLG (TNFSF6)	FAS (TNFRSF6, Apo1)	TNFRSF6b (DcR3)
TNF (TNF-α)	TNFRSF1A (TNFR1)	TNFRSF1B (TNFR2)
TNFSF10 (TRAIL, Apo-2L)	TNFRSF10A (TRAILR1, DR4, Apo2)	TNFRSF10C (TRAILR3, DcR1)
	TNFRSF10B (TRAILR2, DR5)	TNFRSF10D (TRAILR4, DcR2)
		TNFRSF11B (OPG)
